# The value of IL-6, PCT, qSOFA, NEWS, and SIRS to predict septic shock after Percutaneous nephrolithotomy

**DOI:** 10.1186/s12894-024-01502-y

**Published:** 2024-06-07

**Authors:** Yuxin Liu, Qihao Sun, Houtao Long, Zhijian Qiu, Daofeng Zhang, Haiyang Zhang, Ji Chen

**Affiliations:** 1 Department of Urology, Shandong Provincial Hospital, Shandong University, Jinan, 250012 China; 2 Department of Urology, Shandong Provincial Hospital Affiliated to Shandong First Medical University, 324 Jing5 Wei7 Road, Jinan, 250021 Shandong China

**Keywords:** Percutaneous nephrolithotomy, Septic shock, IL-6, PCT, qSOFA

## Abstract

**Background:**

There are numerous methods available for predicting sepsis following Percutaneous Nephrolithotomy. This study aims to compare the predictive value of Quick Sequential Organ Failure Assessment (qSOFA), Systemic Inflammatory Response Syndrome (SISR), National Early Warning Score (NEWS), interleukin-6 (IL-6), and procalcitonin (PCT) for septicemia.

**Methods:**

Patients who underwent percutaneous nephrolithotomy were included in the study and divided into a control group and a septic shock group. The effectiveness of qSOFA, SIRS, NEWS, Interleukin-6, and Procalcitonin was assessed, with Receiver Operating Characteristic curves and Area Under the Curve used to compare the predictive accuracy of these four indicators.

**Results:**

Among the 401 patients, 16 cases (3.99%) developed septic shock. Females, elderly individuals, and patients with positive urine culture and positive nitrite in urine were found to be more susceptible to septic shock. PCT, IL-6, SIRS, NEWS, qSOFA, and surgical time were identified as independent risk factors for septic shock. The cutoff values are as follows: qSOFA score > 0.50, SIRS score > 2.50, NEWS score > 2.50, and IL-6 > 264.00 pg/ml. Among the 29 patients identified by IL-6 as having sepsis, 16 were confirmed to have developed sepsis. The qSOFA identified 63 septicemia cases, with 16 confirmed to have developed septicemia; NEWS identified 122 septicemia cases, of which 14 cases actually developed septicemia; SIRS identified 128 septicemia patients, with 16 confirmed to have developed septicemia. In terms of predictive ability, IL-6 (AUC 0.993, 95% CI 0.985 ~ 1) demonstrated a higher predictive accuracy compared to qSOFA (AUC 0.952, 95% CI 0.928 ~ 0.977), NEWS (AUC 0.824, 95% CI 0.720 ~ 0.929) and SIRS (AUC 0.928, 95% CI 0.888 ~ 0.969).

**Conclusions:**

IL-6 has higher accuracy in predicting septic shock after PCNL compared to qSOFA, SIRS, and NEWS.

## Introduction

Percutaneous nephrolithotomy (PCNL) is the most suitable method for treating large, multiple, or complex kidney stones. Sepsis, as a major complication of PCNL, is one of the most concerning issues for urologists, and its subgroup, septic shock, can lead to increased mortality rates. The incidence of septic shock after PCNL ranges from 0.3 to 9.3% [1, 2], which is 20 times higher than other urological surgeries. In some studies, the mortality rate of septic shock after PCNL has been reported as high as 66% [3]. There are studies that indicate that early identification and intervention in sepsis and septic shock can improve prognosis and reduce mortality rates [4, 5]. Therefore, early identification and treatment of septic shock are crucial for reducing mortality rates and improving outcomes. There are several methods used to assess and predict the occurrence and progression of septic shock, such as the quick sequential organ failure assessment (qSOFA), the systemic inflammatory response syndrome (SIRS), and the National Early Warning Score (NEWS) system. However, the predictive values of these criteria are variable and can be influenced by the underlying diseases of the patients [6]. 

It is well-known that septic shock is a severe systemic inflammatory response. One central event in the pathological and physiological cascade of sepsis is the excessive systemic release of pro-inflammatory cytokines, such as IL-6, in response to microbial invasion [7]. IL-6 is a multifunctional cytokine that regulates immune responses and induces an increase in procalcitonin (PCT) levels [8–10]. Serum levels of IL-6 and PCT are positively correlated with the severity of the inflammatory response [11, 12]. Therefore, when sepsis occurs, IL-6 and PCT serve as biomarkers to monitor the occurrence of septic shock.

There is limited research on early prediction of septic shock after surgery. Our study aims to evaluate the predictive value of these three criteria in the early prediction of septic shock after percutaneous nephrolithotomy, so that clinicians choose appropriate diagnostic tools to avoid catastrophic events.

## Methods

### Study design and patients

This study is a retrospective case-control study conducted at Shandong Provincial Hospital. All 401 patients who underwent PCNL between January 2021 and December 2023 were included in the study. The patients were divided into control group and septic shock group. Diagnosis of SIRS requires meeting at least 2 of the following criteria: body temperature > 38 °C or < 36 °C; heart rate > 90 beats per minute; respiratory rate > 20 breaths per minute or arterial carbon dioxide pressure < 32 mmHg (1 mmHg = 0.133 kPa); white blood cell count > 12 × 10^9^/L or < 4 × 10^9^/L. Severe sepsis refers to infection accompanied by inadequate organ perfusion and/or organ dysfunction (including elevated blood lactate, peripheral circulatory disorders, altered mental status, oliguria, etc.) [13]. Septic shock refers to persistent hypotension despite fluid resuscitation. Sepsis-induced hypotension refers to systolic blood pressure < 90 mmHg or mean arterial pressure < 70 mmHg, or a decrease in systolic blood pressure > 40 mmHg [14]. Basic information of the patients, including age, sex, BMI, comorbidities, surgical history, placement of double-J stent or percutaneous nephrostomy, urine culture results, and urinary nitrite were collected through the hospital data system.

### Surgical procedure

Following general anesthesia, precise localization of the kidney stone was achieved with the assistance of ultrasound imaging. Subsequently, the stone was accurately punctured using a needle, through which a hook-shaped guide wire was inserted. Carefully, a dilator was advanced along the guide wire and expanded to its maximum capacity. After optimal dilation was achieved, the stone was fragmented and extracted using a holmium laser fiber in conjunction with pneumatic ballistic technology. Subsequently, under the guidance of the guide wire, the fragmented stone particles were grasped and removed with a stone retrieval basket. This meticulous and precise approach ensured the successful extraction of the kidney stone.

### IL-6, PCT, qSOFA, NEWS, and SIRS assessment

To facilitate early detection, blood samples were obtained two hours post-surgery. The qSOFA score ranges from 0 to 3 and includes altered mentation, systolic blood pressure of 100 mm Hg or less, and respiratory rate of 22/min or greater as criteria [15]. The evaluation of septic shock was conducted utilizing the SIRS scoring criteria outlined in Sepsis-2, encompassing parameters such as heart rate, mean arterial pressure (MAP), respiratory rate, arterial oxygen pressure, body temperature, white blood cell count, blood glucose levels, and consciousness [14]. The NEWS score, ranging from 0 to 20, relied on factors including respiratory rate, oxygen saturation, supplemental oxygen requirement, body temperature, systolic blood pressure, pulse rate, and level of consciousness [16]. The normal range for IL-6 is 0–7 pg/ml, with values exceeding 7 pg/ml indicative of an inflammatory response [8, 17]. Similarly, the normal range for PCT is 0-0.5 ng/ml, and levels surpassing 0.5 ng/ml signify an inflammatory reaction [18]. 

### Statistical analysis

All data were analyzed using SPSS 18.0. Continuous variables were compared using Student’s t-test and the Mann-Whitney test, and categorical variables were compared using the chi-square test. Logistic regression was used to determine independent risk factors when the P-value of two variables was less than 0.05 (*P* < .05). The predictive ability of IL-6, PCT, NEWS, and SIRS was evaluated using the area under the receiver operating characteristic (ROC) curve (AUC) values.

## Results

### Clinical characteristics of patients

The clinical characteristics of the patients were presented in Table 1. All patients underwent PCNL surgery within the past 3 years, with 247 (61.6%) being male and 154 (38.4%) being female. Postoperative septic shock occurred in 16 cases (3.99%), while 385 cases (96.01%) did not develop septic shock. The mean age was 51.24 ± 12.03 years, and the mean body mass index (BMI) was 25.79 ± 3.52. Approximately 217 participants (54.1%) had comorbidities such as hypertension, diabetes, heart disease, stroke, and chronic kidney disease.

**Table 1:** Baseline characteristics and clinical data of included patients

Variable	Septic shock (*n* = 16)	No septic shock (*n* = 385)	*p* value
Number of punctures (n, %)			1.000^a^
1	14/16 (87.5%)	336/385 (87.3%)	
≥ 2	2/16 (12.5%)	49/385 (12.7%)	
Operative time (n, %)			0.044
< 90	4/16 (25.0%)	195/385 (50.6%)	
≥ 90	12/16 (75.0%)	190/385 (49.4%)	
NEWS score (mean ± SD)	3.75 ± 1.57	2.36 ± 0.69	0.003
PCT [ng/mL, mean ± SD]	4.64 ± 11.51	0.11 ± 0.60	0.137
IL-6 [pg/mL, mean ± SD] qSOFA	5321.59 ± 6862.21 0 (0–0)	72.89 ± 342.93 1 (1–2)	0.008 <0.001
SIRS score (mean ± SD)	3.81 ± 0.54	1.95 ± 1.04	< 0.001
Pyonephrosis (n, %)	7/16 (43.8%)	12/385 (3.1%)	< 0.001^a^
Decrease in Hb (n, %)	12/16 (75.0%)	300/385 (77.9%)	0.762^a^
Increase in Cr (n, %)	13/16 (81.3%)	311/385 (80.8%)	1.000^a^

NEWS the National Early Warning score (NEWS), PCT procalcitonin, IL-6 Interleukin 6, the quick Sepsis-related Organ Failure

Assessment (qSOFA), the Systemic Inflammatory Response Syndrome (SIRS)

### Univariate analysis

The results were shown in Tables 1 and 2. There were statistically significant differences (*P* < .05) between the septic shock group and the control group in terms of gender, positive urine culture, and positive urine nitrite. However, there were no statistically significant differences (*P* > .05) between the two groups in terms of surgical approach, underlying diseases, and surgical history.

**Table 2:** Results of a univariate analysis comparing the control group and the septic shock group

Variables	All patients (*N* = 401)	Septic shock group (*n*_1_ = 16)	Control group (*n*_2_ = 385)	χ2/t	*P*
Male (n, %)	247 (61.6%)	3 (18.8%)	244 (63.4%)	12.933	< 0.001
Age (years, mean ± SD)	51.24 ± 12.03	53.31 ± 8.65	51.15 ± 12.16	0.704	0.482
BMI (Kg/m^2^, mean ± SD)	25.79 ± 3.52	26.47 ± 3.70	25.76 ± 3.53	0.784	0.434
Comorbidities (n, %)					
Hypertension	134 (33.4%)	6 (37.5%)	128 (33.2%)	0.125	0.724
Diabetes mellitus	59 (14.7%)	3 (18.8%)	56 (14.5%)		0.716^a^
Cardiovascular disease	19 (4.7%)	1 (6.3%)	18 (4.7%)		0.547^a^
Chronic kidney disease	5 (1.2%)	2 (12.5%)	3 (0.8%)		0.014^a^
History of urolithiasis surgery (n, %)					
PCNL	84 (21.0%)	4 (25.0%)	80 (20.8%)		0.753^a^
URS	58 (14.5%)	1 (6.3%)	57 (14.8%)		0.487^a^
EWL	63 (15.7)	1 (6.3%)	62 (16.1%)		0.485^a^
Open surgery	26 (6.5%)	1 (6.3%)	25 (6.5%)		1.000^a^
No	218 (54.4%)	7 (43.8%)	211 (54.8%)	0.757	0.384
Preoperative drainage (n, %)					
Ureteral double-J stenting	73 (18.2%)	4 (25.0%)	69 (17.9%)		0.506^a^
Percutaneous nephrostomy	47 (11.7%)	1 (6.3%)	46 (11.9%)		0.706^a^
Positive history of urine culture (n, %)	150 (37.4%)	14 (87.5%)	136 (35.3%)	17.860	< 0.001
Positive history of urinary nitrite (n, %)	48 (12.0%)	10 (62.5%)	38 (9.9%)		< .001^a^

^a^ Fisher’s Exact Test

### Multivariable analysis

The multivariable analysis encompassed variables such as the number of channels, surgical duration, presence of pyonephrosis, levels of PCT and IL-6, qSOFA criteria, SIRS criteria, NEWS score, as well as changes in hemoglobin and serum creatinine levels (Table 3). Findings revealed that IL-6, qSOFA, SIRS, NEWS, and pyonephrosis, emerged as independent risk factors (*P* < .05). Conversely, PCT failed to exhibit a statistically significant difference between the septic shock and control groups (*P* = .137). Moreover, the number of channels utilized, alterations in hemoglobin levels, surgical duration, and variations in serum creatinine levels were not identified as independent risk factors.

**Table 3:** Multivariate analysis results between septic shock group and control group

Variables	β	SE	Waldχ^2^	OR	95% CI	*P*
Male	-2.345	1.763	1.782	0.095	0.003–3.010	0.182
History of urolithiasis surgery	-0.866	1.460	0.352	0.421	0.024–7.362	0.553
Positive history of urine culture	2.584	2.547	1.029	13.250	0.090-1950.1	0.310
Positive history of urinary nitrite	-3.074	2.106	2.130	0.046	0.001–2.869	0.144
Operation time	0.019	0.015	1.650	1.019	0.990–1.048	0.199
NEWS score	-1.649	0.747	4.871	0.192	0.044–0.831	0.027
IL-6 qSOFA score	-0.002 -2.780	0.001 1.400	6.948 3.942	0.998 0.062	0.997–0.999 0.004–0.965	0.008 0.047
SIRS score	-1.751	0.889	3.877	0.174	0.030–0.992	0.049
pyonephrosis	-4.783	2.288	4.369	0.008	0.000-0.742	0.037

Standard error (SE), odds ratio (OR), confifidence interval (CI), the quick Sepsis-related Organ Failure Assessment

(qSOFA), the National Early Warning score (NEWS), interleukin 6 (IL-6), the Systemic Inflammatory Response Syndrome (SIRS)

### Optimal cutoffs of IL-6, PCT, qSOFA, SIRS, and NEWS

**Fig. 1:**
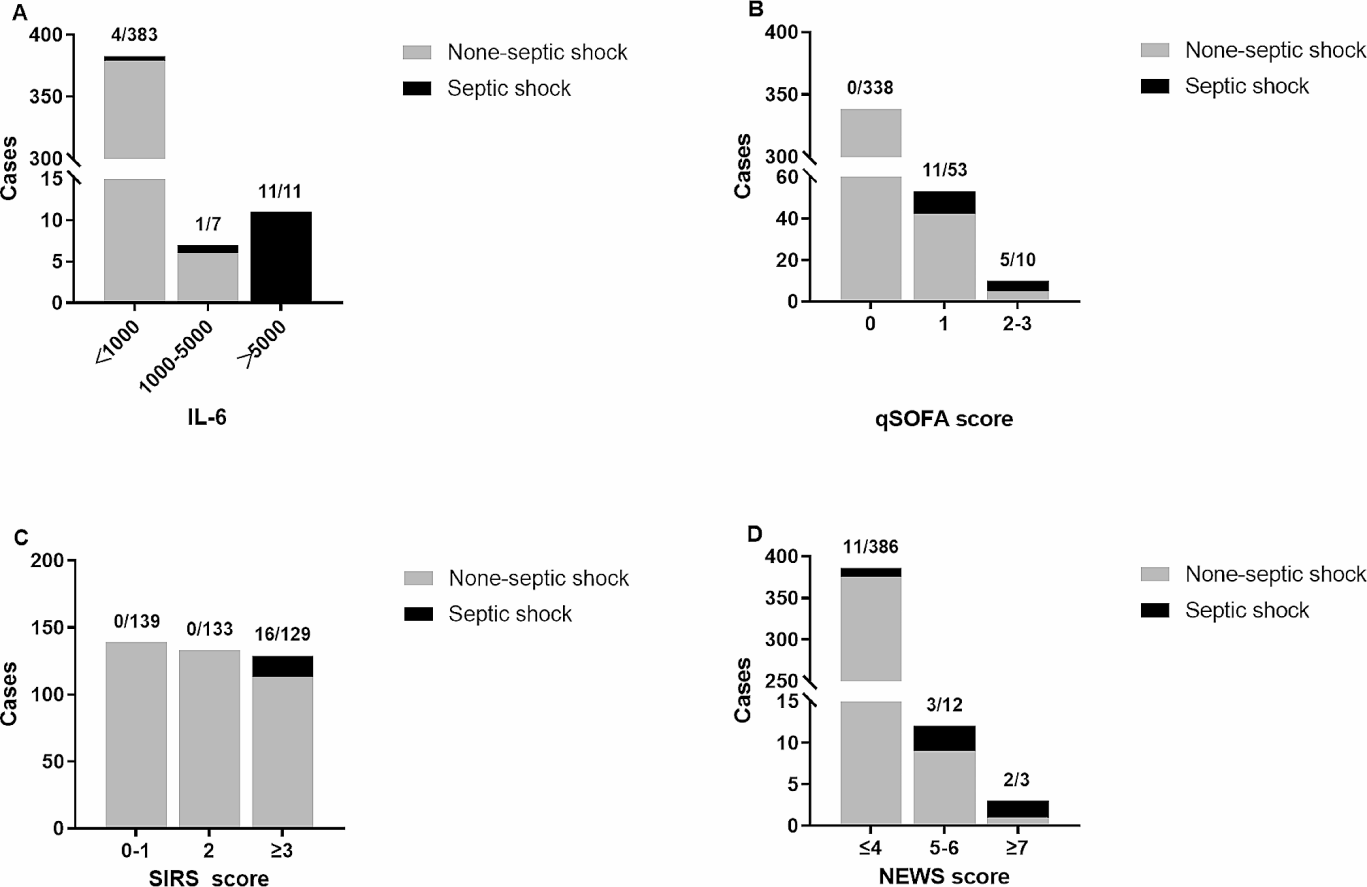
Distribution of included cases according to the number of (**A**) IL-6, (**B**) qSOFA, (**C**) SIRS, (**D**) NEWS criteria met and corresponding septic shock rates

The qSOFA scores were divided into three categories: 1, 2, and 3, comprising 338 (84.3%), 53 (13.2%), and 10 (2.5%) cases in each respective group. The distribution of SIRS scores resulted in three groups: 0–1, 2, and > 2 (3–4), comprising 139 (34.7%), 133 (33.1%), and 129 (32.1%) cases in each respective group. NEWS scores were categorized into three groups: ≤ 4, 5–6, and ≥ 7, with 368 (91.8%), 12 (3.0%), and 3 (0.7%) cases in each group, respectively. IL-6 levels were stratified into three groups: < 1000 pg/ml, 1000–5000 pg/ml, and > 5000 pg/ml, with 383 (95.5%), 7 (1.7%), and 11 (2.7%) cases in each group, respectively. (Table 4). The histogram (Fig. 1) shows the proportions of each section of the three criteria while the proportions of septic shock patients were also shown. High IL-6 value, qSOFA score, NEWS score, and SIRS score were more inclined to present septic shock.

**Table 4:** Clinical utility indexes of IL-6, qSOFA score, NEWS score and SIRS score for predicting septic shock after PCNL

	Optimal cut-off	Sensitivity (%)	Specificity (%)	Youden index
IL-6	264.00	100.00	96.60	0.966
qSOFA score	0.50	100.00	87.80	0.878
NEWS score	2.50	87.50	71.90	0.594
SIRS score	2.50	100.00	70.60	0.706

The quick Sepsis-related Organ Failure Assessment (qSOFA), the National Early Warning score (NEWS), interleukin 6 (IL-6), Systemic Inflammatory Response Syndrome (SIRS)

**Fig. 2:**
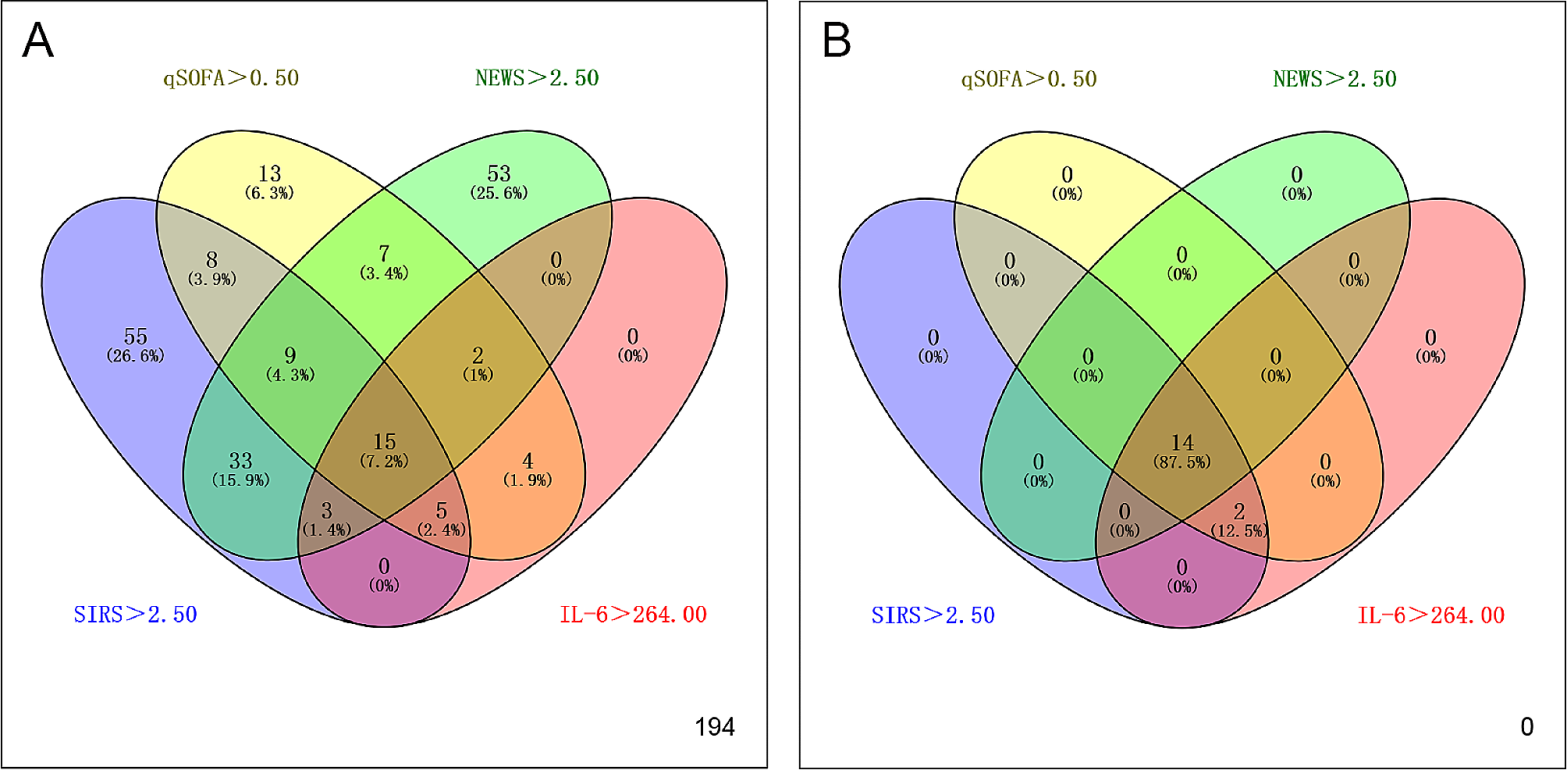
The distribution diagram of (**A**) all patients identified as septic and (**B**) patients with septic shock

Criteria of > 0.5 in qSOFA, > 2.50 in SIRS, > 2.50 in NEWS and ≥ 264.00 pg/ml in IL-6 were set as optimal cutoffs to predict post-PCNL septic shock. Figure 2A showed the distribution of 194 patients who at least had met one of these criteria but failed to develop septic shock. Among the 16 patients who developed septic shock, 14 of them met the criteria for septic shock according to the NEWS scoring system, and all patients met the predictive values for septic shock based on qSOFA, SIRS, and IL-6 (Fig. 2B).

### The predictive value of IL-6, qSOFA, SIRS, and NEWS

**Fig. 3:**
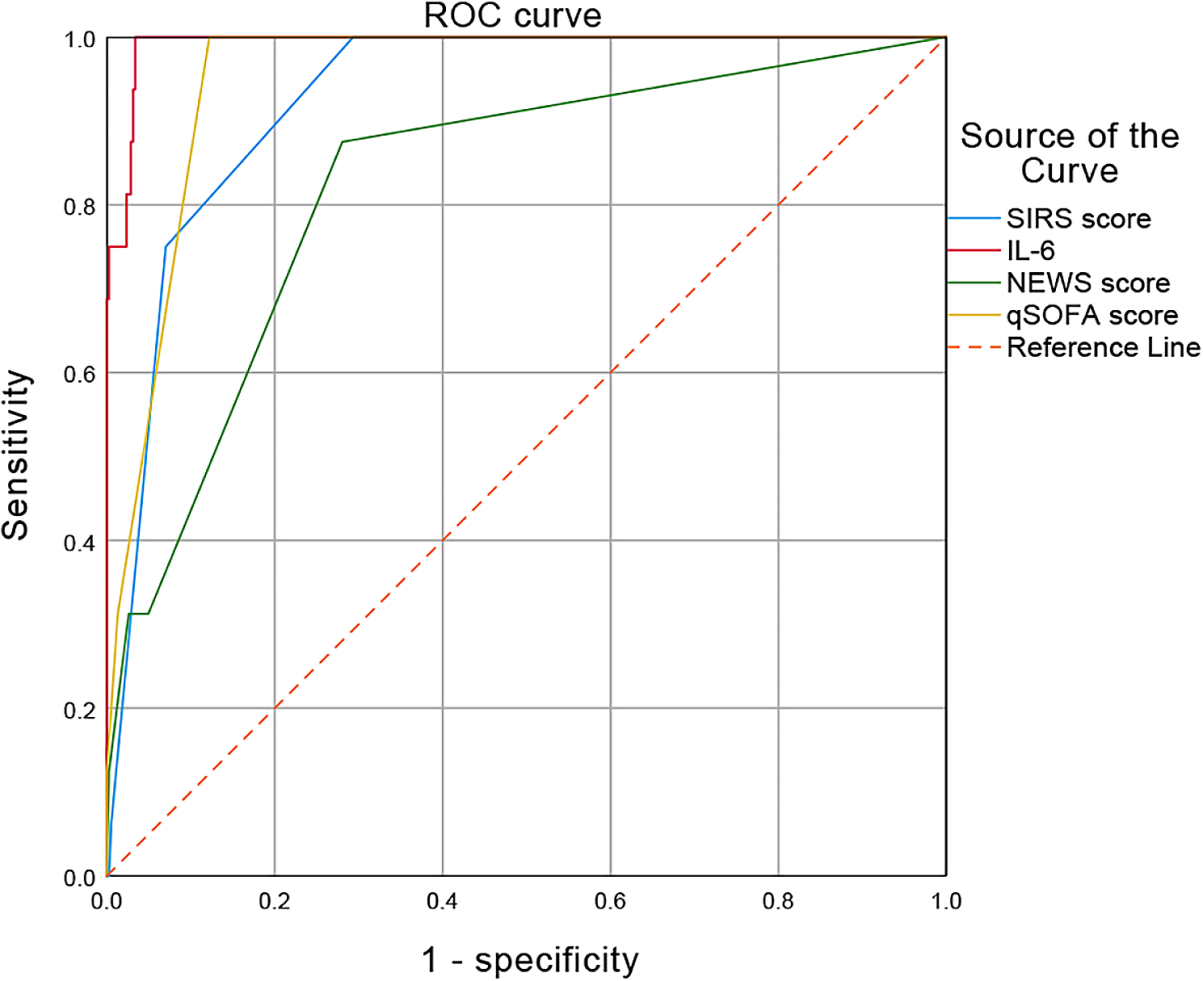
Comparison of the AUC of qSOFA, SIRS, NEWS, and IL-6 criteria for septic shock

In this study, the predictive values of IL-6, qSOFA, SIRS, and NEWS were compared using the ROC curve (Fig. 3). The AUC of IL-6 was 0.993 (95% CI 0.985-1). The qSOFA demonstrated an AUC of 0.952 (95% CI 0.928–0.977), SIRS showed an AUC of 0.928 (95% CI 0.888–0.969), and NEWS exhibited an AUC of 0.824 (95% CI 0.720–0.929). IL-6 exhibited significantly higher AUC values compared to qSOFA (Z = 4.186, *P* < .001), NEWS (Z = 3.309, *P* = .001), and SIRS (Z = -3.419, *P* = .001). This indicated that IL-6 had a higher predictive value.

## Discussions

Sepsis emerges as a significant contributor to morbidity and mortality post-PCNL, particularly affecting elderly, immunocompromised, and critically ill individuals [4, 19, 20]. The prevalence of septic shock following PCNL has shown a notable rise over the past years, escalating from 1.2% in 1999 to 2.4% in 2009, marking a twofold increase within a 17-year period [21]. Studies have underscored the pivotal role of prompt recognition and proactive management of sepsis and septic shock in enhancing patient outcomes and diminishing mortality rates [4, 20, 22]. 

In 1991, the American College of Chest Physicians (ACCP) and the Society of Critical Care Medicine (SCCM) convened a consensus meeting, introducing the concepts of “systemic inflammatory response syndrome” (SIRS), “sepsis,” and “septic shock” based on clinical and laboratory parameters [4, 5, 20, 23]. SIRS has been widely used in various medical institutions, but its broad inclusion criteria, low specificity, and potential for overdiagnosis have led to the proposal of different scoring systems [6, 24]. Among them, the National Early Warning Score (NEWS) is more widely applied. However, due to different hospitals having different evaluation criteria for accurate issues, there is a lack of equal communication of patient conditions between different medical institutions, and there is also no unified training for healthcare personnel [12, 16]. In order to establish a standardized system, NEWS was proposed in 2012 based on the NHS Early Warning Score. It has since been widely used in ICUs, emergency departments, and other medical settings to assess patients general conditions and determine whether they need escalated medical care and nursing [16]. Nevertheless, the efficacy of NEWS in improving patient care lacks empirical support, with existing studies being predominantly observational and establishing a correlation between score deterioration and prognosis, as well as early detection of patient deterioration. Notably, the accuracy of postoperative NEWS assessment on the day of surgery is compromised by surgical stress and intraoperative medication use [6]. 

In addition, qSOFA scores are widely used in the identification of sepsis patients. qSOFA scores are a simplified version of the SOFA score, which evaluates six organs or systems – respiratory, coagulation, liver, cardiovascular, central nervous, and renal – by quantifying the function of each organ with scores [15]. The qSOFA score quickly identifies septic patients by assessing blood pressure, respiratory rate, and state of consciousness [15]. Nevertheless, the qSOFA score is constrained by its low sensitivity. Two meta-analyses have revealed that its ability to predict mortality in suspected infection patients was below 50% [25, 26]. This lack of sensitivity implies that a significant proportion of high-risk sepsis patients may go undetected, hampering timely intervention for sepsis [27]. 

One central event in the pathophysiological cascade of sepsis is the excessive systemic release of pro-inflammatory cytokines in response to microbial invasion, such as IL-6 [7, 8]. IL-6 is a multifunctional cytokine that regulates immune responses and is considered an endogenous pyrogen that causes fever in infected patients [8, 28]. After infection and inflammation occur, IL-6 is generated first and its levels rapidly increase, reaching a peak within 2 h [8, 17]. The elevated levels of IL-6 are consistent with the severity of infection and induce the increase of PCT and C-reactive protein (CRP) levels, which start to rise 2 h and 6 h after infection, respectively [7, 10, 29]. The serum PCT value reaches its peak 12 to 24 h after infection [10, 30, 31]. As an upstream inflammatory factor of PCT, IL-6 has a more timely response, and the studies have shown that high levels of IL-6 indicate poor prognosis in septic patients. However, due to the unclear timing of inflammatory triggers in departments such as the ICU, the use of IL-6 in predicting infections is somewhat limited.

This study considers surgery as an inflammatory trigger and measures serum IL-6 levels two hours after surgery. At this time, the inflammation is in the early stages, making it possible to predict sepsis in the early stages of infection. Additionally, IL-6 is a laboratory marker that provides rapid and relatively objective results, with high sensitivity. This study evaluated the predictive value of three scoring systems by measuring their ROC curves and AUC values. The value of AUC for IL-6 was 0.993, for SIRS was 0.928, and for NEWS was 0.824. The AUC for IL-6 was statistically different from the AUC for SIRS and NEWS. Compared to NEWS and SIRS, IL-6 was less affected by surgical stress and intraoperative medications, and had higher specificity in prediction.

There were two main limitations of this study. First, it was a single institutional retrospective study design. Since all the data were from clinical sources, there may be potential bias. Second, the sample size of this study was small. In the next study, we will expand the sample size and further confirm the conclusions through prospective studies.

## Conclusion

IL-6 has a high predictive value in the early postoperative prediction of sepsis. Its application in clinical practice can help identify sepsis in a timely manner and intervene with treatment, thereby reducing the occurrence of septic shock and mortality. Furthermore, compared to qSOFA, SIRS, and NEWS scores, IL-6 has higher specificity. IL-6 has higher accuracy in predicting septic shock after PCNL compared to qSOFA, SIRS, and NEWS. It is necessary to make a comprehensive selection in clinical applications.

## Data Availability

The datasets used and analysed during the current study are available from the corresponding author on reasonable request.

## Abbreviations

IL-6: Interleukin-6
NEWS: National Early Warning Score
PCT: Procalcitonin
PCNL: Percutaneous nephrolithotomy
SISR: Systemic Inflammatory Response Syndrome
